# Effect of prenatal administration of low dose antibiotics on gut microbiota and body fat composition of newborn mice

**DOI:** 10.3164/jcbn.17-53

**Published:** 2017-12-29

**Authors:** Ayumi Yoshimoto, Takashi Uebanso, Mutsumi Nakahashi, Takaaki Shimohata, Kazuaki Mawatari, Akira Takahashi

**Affiliations:** 1 Department of Preventive Environment and Nutrition, Institute of Biomedical Sciences, Tokushima University, 3-18-15 Kuramoto, Tokushima 770-8503, Japan; 2 Graduate School of Technology, Industrial and Social Sciences, Tokushima University, 3-18-15 Kuramoto, Tokushima 770-8503, Japan

**Keywords:** transgenerational, maternal, adiposity, DGGE, CE-MS

## Abstract

Several environmental factors during the prenatal period transgenerationally affect the health of newborns in later life. Because low-dose antibiotics have been used for promoting the growth of crops and livestock in agriculture, humans may have ingested residual antibiotics for several decades. However, the effect of prenatal administration of low-dose antibiotics on newborns’ health in later life is unclear. In the present study, we found that prenatal treatment of murine mothers with low-dose antibiotics increased the abundance of bacteria of the phylum *Firmicutes* and the genera *Clostridium IV* and *XIVa* in feces from pups. In addition, the body fat percentage of mice in the antibiotic-treated group was higher than those in the control group at 12 weeks of age even though all pups were fed a standard diet. The body fat percentage of all mice was correlated with the abundance of fecal bacteria of *Clostridium IV* and *XIVa*. These results predict that low-dose antibiotic administration during the prenatal period affects the gut microbiota of newborns and possibly their health in later life.

## Introduction

Human health is influenced by both genetic and environmental factors.^(1)^ Maternal environmental factors, especially during the prenatal period, affect children’s future health.^(2)^ For example, maternal malnutrition increases the risks of obesity, diabetes and psychiatric disorders in children,^(3)^ and maternal smoking increases the risk of low birth weight and developmental disorders.^(4)^ In addition, alcohol intake during pregnancy can affect the cognitive behavior of the child through childhood and adolescence,^(5)^ and cocaine use during pregnancy induces hypoxia and growth disturbances in children.^(6)^

Antibiotics have been used in the agricultural setting to promote the growth of animals and plants as well in the medical setting to prevent and treat infectious diseases.^(7)^ Nonetheless, antibiotic use during the perinatal period and disturbance of gut microbiota in early life due to cesarean delivery possibly increases the risk of body weight gain in early childhood.^(8)^ Furthermore, administration of low-dose antibiotics to weaning mice was found to increase body fat mass and change gut microbiota and gene expression.^(9,10)^ These findings suggest that the intake of antibiotics in the postnatal or weaning periods affects host gut microbiota and metabolism. However, it has been unclear how antibiotic exposure in the mother during the fetal period affects the future health of the child. In this study, we found that low-dose antibiotic treatment of female mice from 1 week before pregnancy to birth disturbed the gut microbiota of the pups and that increased amounts of *Clostridium IV* and *XIVa* in feces correlated with increased body fat percentage in pups.

## Materials and Methods

### Animals

We purchased six female C57BL/6J mice on day 7 of gestation from a local breeding colony (Charles River, Yokohama, Japan). After birth, twelve female pups were divided into the control group (*n* = 6) and antibiotics (Ab) group (*n* = 6) at 7 weeks of age. Mice in the Ab group were administered a mixture of three different kinds of antibiotics at subtherapeutic levels: penicillin V (Tokyokasei, Tokyo, Japan), chlortetracycline (Sigma, St. Louis, MO) and vancomycin (WAKO, Osaka, Japan) per day at 1 µg/g body weight^(10)^ from 7 weeks of age. The mice in the control group were administered a vehicle. After 7 days of administration of antibiotics or vehicle, mice were mated with male mice for 4 days. Nine female mice were pregnant (control group, *n* = 5; Ab group, *n* = 4). Administration of antibiotics or vehicle continued until the time of birth. Fecal samples were collected before and after administration of antibiotics and stored at −80°C until analysis. Pups were co-housed with the mothers until weaning. After weaning, pups were fed a standard diet (MF; Oriental Yeast Co., Tokyo, Japan) and tap water *ad libitum*. Mice were housed in cages maintained at constant temperature (23 ± 2°C) and humidity (65–75%) with a 12-h light (8:00–20:00), 12-h dark (20:00–8:00) cycle. During the experimental period, their body weights were measured weekly. At 8 weeks old, their fecal samples were collected and stored at −80°C. At 12 weeks old, their body fat percentage was analyzed by CT. At 13 weeks old, all pups were dissected, and liver, serum, cecum and fecal samples were collected and rapidly stored at −80°C. The University of Tokushima Animal Use Committee approved the study (T14010), and mice were maintained according to the National Institutes of Health Guidelines for the Care and Use of Laboratory Animals.

### Fecal bacteria analysis by quantitative real-time PCR

DNA was extracted from fecal samples using the QIAamp DNA Stool Mini Kit (QIAGEN, Tokyo, Japan). The extracted DNA concentration was adjusted to 10 ng/µl, and the relative abundance of total bacteria and specific bacteria [phylum or genus, including *Firmicutes*, *Bacteroidetes*, *Lactobacillus*, *Clostridium IV*, *Clostridium XIVa* and *Bacteroides* (Table 1)] was measured using quantitative real-time PCR using SYBR Premix Ex Taq (TaKaRa, Otsu, Japan).

### Fecal bacterial analysis by denaturing gradient gel electrophoresis

Denaturing gradient gel electrophoresis (DGGE) analysis was carried out using the DCode^TM^ Universal Mutation Detection System according to the manufacturer’s instructions (Bio-Rad Labs, Hercules, CA). The specific region of the 16S rRNA genes of all bacteria (GC-*Eubacteria*, positions 341 to 518 in *Escherichia coli*) and specific phyla (GC-*Firmicutes*, positions 934 to 1060; GC-*Bacteroidetes*, positions 934 to 1060) in fecal DNA was amplified by KOD FX Neo (TOYOBO, Osaka, Japan) (Table 1). The denaturing gradient was formed with 6% (for all bacteria) or 8% (for specific phyla) acrylamide (acrylamide-bis 37.5:1) with the denaturing gradient ranging from 20% to 80% (for all bacteria) or 50% to 60% (for specific phyla) for analysis of amplified 16S rRNA fragments. PCR products were electrophoresed at 200 V for 4 h. After electrophoresis, gels were stained with Gelstar (Lonza Japan, Tokyo, Japan) for 30 min and analyzed by Chemi-Doc image analysis equipment (Bio-Rad Labs). Image Lab software, ver. 5.0 (Bio-Rad Labs) was used for identification of bands and normalization of band patterns from DGGE gels.

### Measure of body fat percentage by CT

The body fat percentage of male mice was measured using X-ray computed tomography (CT) Latheta LCT-200 for experimental animals (Hitachi Aloka, Tokyo, Japan). Mice were anesthetized with isoflurane (Abbott Japan Co., LTD, Tokyo, Japan), fixed to Latheta and abdominal part L4–L5 was measured. Measurement conditions were: rotational speed, standard; slice thickness, 192 µm × 107 sheet; voxel size, 96 × 192 µm; x-ray tube voltage, low; shooting conditions, 48 mm view, 180°, 436, asynchronous. Filmed images were analyzed by computer, and body fat percentage was calculated.

### Metabolome analysis of cecal contents

Cecal contents from 13 weeks old mice which stored at −80°C were weighed and completely homogenized in 500 µl methanol containing 50 µM methionine sulfone and camphor-10-sulfonic acid as internal standards. The homogenates were mixed with 500 µl chloroform and 200 µl Milli-Q water. Samples were centrifuged (2,300 *g*, 5 min, 4°C), and then the supernatant was centrifugally filtered using 5-kDa cut-off filters (Millipore, Bedford, MA) until all was filtered (9,100 *g*, 4°C). The filtrate was centrifugally concentrated in a vacuum evaporator, dissolved in Milli-Q water and analyzed by capillary electrophoresis electrospray ionization time-of-flight mass spectrometry (CE-TOFMS).

CE-TOFMS analysis was performed by an Agilent CE system combined with a TOFMS (Agilent Technologies, Palo Alto, CA) as reported by Human Metabolome Technologies Inc. (HMT, Tsuruoka, Japan).^(11,12)^ Each metabolite was identified and quantified based on the peak information including *m/z*, migration time and peak area.

### Statistical analysis

The mean and standard deviation were calculated for all results. The *t* test or Mann-Whitney *U* test (*p*<0.05) was used to compare two groups. Principal component analysis (PCA) was carried out with Excel Tokei 2010 (SSPZ) and Mass Profiler Professional software (Agilent Technology).

## Results

**Fig. 1:**
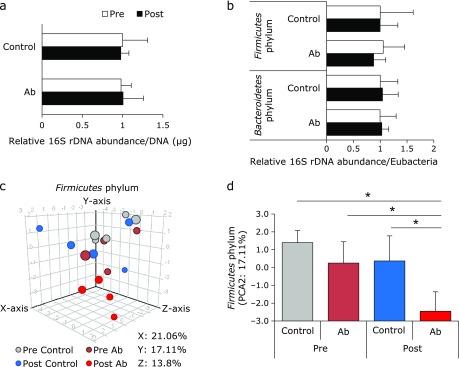
Fecal microbiota of the mother mice. (a) The relative amounts of mothers’ total fecal bacteria before antibiotic administration (pre), and after antibiotic administration (post) were analyzed by RT-PCR for *Eubacteria* (as total bacteria) and the phyla *Bacteroidetes* and *Firmicutes*. Total bacteria were corrected for fecal DNA concentration. *Bacteroidetes* and *Firmicutes* were corrected for the amounts of total bacteria. (c) Three-dimensional of PCA plot of DGGE band pattern in *Firmicutes* phylum. (d) Scale of Y axis in PCA of *Firmicutes* phylum. Control group (*n* = 5), Antibiotics group (Ab, *n* = 4). Data are shown as average and SD. *p<0.05.

Cho *et al.*^(10)^ reported that low dose antibiotics (1 µg/g BW/day of penicillin V, vancomycin, and chlortetracycline) administration in early life alters the adiposity in mice. To investigate the effects of the administration of low-dose antibiotics to pregnant mothers on the future health of their pups, we exposed female mice to penicillin V, chlortetracycline and vancomycin from 1 week before pregnancy to birth. During the exposure period, changes in the mothers’ body weight (25.0 ± 0.9 g in the control group and 24.8 ± 1.1 g in the Ab group) and water consumption (3.6 ± 0.4 g/g BW/day in the control group and 3.6 ± 0.5 g/g BW/day in the Ab group) did not differ between the control and Ab groups. Low-dose antibiotic administration did not affect the number of pups (mean 7.2 ± 1.9 in the control group and 5.6 ± 0.8 in the Ab group), the ratio of males to females (control group, 48%:52%; Ab group, 59%:41%). We could not define any differences between the relative amounts of total bacteria, *Firmicutes* or *Bacteroidetes* in the mothers’ feces before and after antibiotic administration (Fig. 1a, b). In order to analyze the mother’s gut microbiota in more detail, we conducted DGGE analysis targeting *Eubacteria* (as total bacteria), *Firmicutes* and *Bacteroidetes*. Band patterns from amplified DNA (16s RNA V2 to V3 region) (Fig. 1 and Supplemental Fig. 1*) and PCA (Fig. 1 and Supplemental Fig. 1*) indicated that the compositions of fecal microbiota were not significantly different between the control and Ab groups. We analyzed the *Firmicutes* phylum by three-dimensional PCA, which clearly showed differences among the post-Ab group and other groups (Fig. 1c, d). Although the composition of gut microbiota was heterogeneous (Supplemental Fig. 1*), the individual distance of the *Firmicutes* in the Ab group was significantly lower than the pre-treatment Ab group and the pre- and post-control group (Fig. 1h and Supplemental Fig. 1*). These results suggest that low-dose antibiotics affect the bacterial composition of the mothers’ feces, especially bacteria in the phylum *Firmicutes*.

**Fig. 2:**
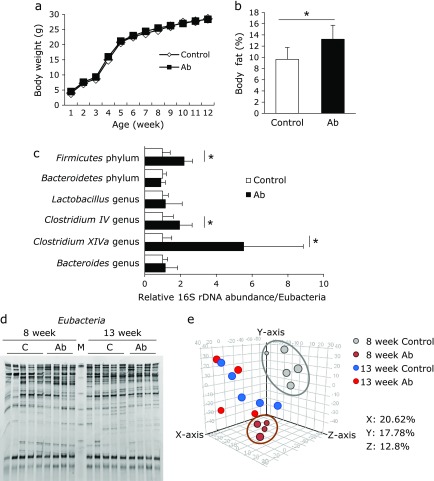
Fecal microbiota and body composition in mouse pups. (a) Changes in body weight of pups from weeks 1 to 12. (b) Percentage of abdominal body fat at 12 weeks. (c) The relative amounts of pups’ fecal bacteria at 8 weeks was analyzed by RT-PCR. The number of all bacteria was corrected by the amount of *Eubacteria*. (d) Band image of DGGE analysis of DNA from feces at 8 and 13 weeks. (e) PCA plots of each DGGE band pattern in total bacteria. M, DNA marker; Control group (*n* = 5); Antibiotics group (Ab, *n* = 4). Data are shown as average and SD. *p<0.05.

**Fig. 3:**
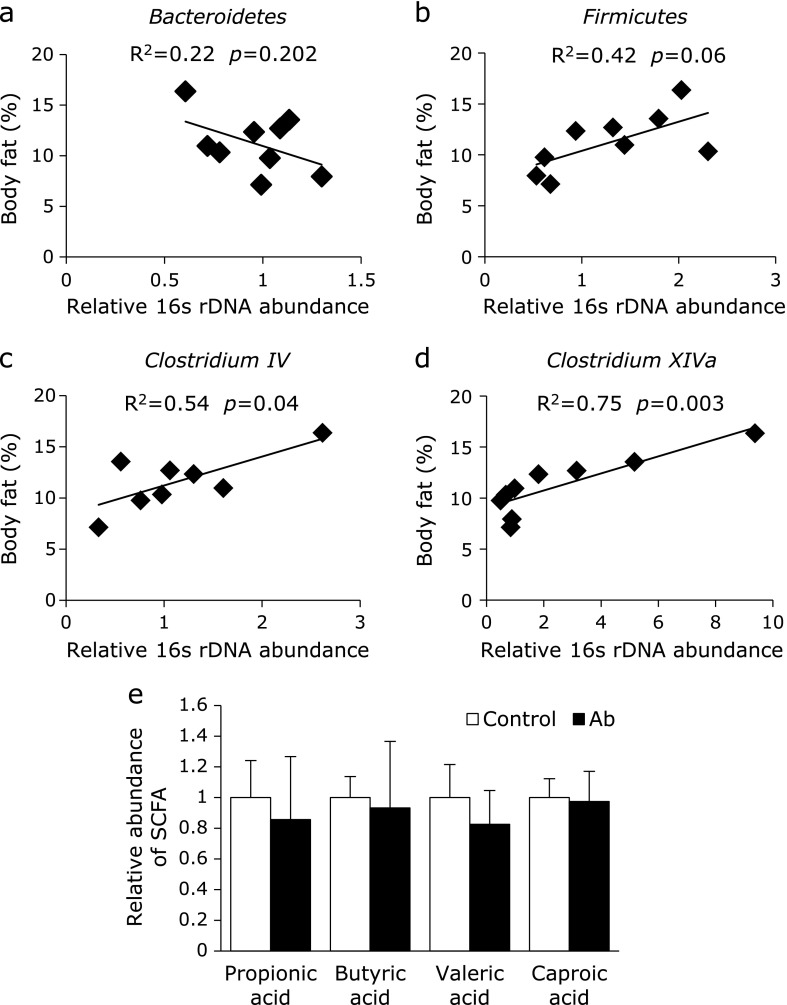
Correlation between fecal microbiota and percentage of body fat and the concentration of luminal short chain fatty acids in pups. (a–d) Relationship between the abundance of fecal bacterial groups and percentage of body fat in pups. Data were analyzed using Spearman’s rank correlation test. Control group (*n* = 5); Antibiotics group (Ab, *n* = 4). (e) Changes in the relative concentration of luminal short chain fatty acids (SCFA) at 13 weeks old. Control group (*n* = 4); Antibiotics group (Ab, *n* = 4). Data are shown as average and SD.

Next, we analyzed the body composition and gut microbiota of the pups. Body weight was not significantly different over time between pups whose mothers had received antibiotics or the control (Fig. 2a). However, pups in the Ab group had a significantly higher abdominal body fat percentage at 12 weeks compared with the control group (Fig. 2b). The number of bacteria from the phylum *Firmicutes* and genera *Clostridium IV* and *XIVa* were significantly higher in the Ab group than those in the control group (Fig. 2c). According to PCR-DGGE analysis for total bacteria, PCA and hierarchical clustering of the gut microbiota of the pups at 8 weeks was different between the two groups (Fig. 2d, e and Supplemental Fig. 2*). These differences in the gut microbiota of the pups were reduced at 13 weeks. A similarity of the gut microbiota between mother and pups (at 8 weeks) were not different between the two group (Supplemental Fig. 3*). However, there is a limitation of this results that individual variances of the composition of gut microbiota were remarkable in the much pairs. We then analyzed the association between body fat percentage and gut microbiota. There was no correlation between body fat percentage and the abundance of *Bacteroidetes* and *Firmicutes*, but the abundance of *Clostridium IV* and *XIVa* were significantly positively correlated with body fat percentage (Fig. 3a–d).

Finally, we analyzed luminal metabolites in the pups by CE-MS. Based on their *m/z* values and migration times, 160 metabolites were measured in the luminal contents. There was no metabolite that was significantly different between the two groups, including short-chain fatty acids (SCFA) (Fig. 3e).

## Discussion

In the present study, we found that administration of low-dose antibiotics from 1 week before pregnancy to birth affected the gut microbiota and abdominal fat percentage of the pups but not body weight. The changes in gut microbiota, especially in *Firmicutes* and *Clostridium*, could be observed at 8 weeks of age. In addition, the genera *Clostridium IV* and *XIVa* were correlated with increased abdominal fat percentage.

Cho *et al.*^(10)^ reported that antibiotic treatment of pups at subtherapeutic levels (penicillin, vancomycin, chlortetracycline, at 1 µg/g body weight per day) for 7 weeks beginning at the time of weaning increased the relative concentrations of fecal *Firmicutes* compared to *Bacteroidetes*, which accompanied the observed increases in adiposity with body weight gain. The effects of low-dose antibiotics on the adiposity of pups may depend on the timing of antibiotic administration. Treatment with low-dose penicillin from 1 week prior to birth to 20 weeks resulted in increased body fat and body weight,^(9)^ while the present study showed that administration of low-dose antibiotics only to mothers prior to birth promoted the deposition of energy as abdominal fat without obesity. Because these metabolic changes were emphasized by a high fat diet after weaning,^(9)^ we should carefully select the low-dose antibiotics or antibiotic dietary compounds that are exposed to the mothers.

We found that there are large individual differences in intestinal bacterial flora. Gut microbial communities vary even within adult monozygotic and dizygotic twin pairs.^(13)^ Moreover, Andoh *et al.*^(14)^ reported that obesity associated gut microbiota (higher amount of the order *Clostridiales*) in the Japanese population was different from that in western people. Some environmental factors including diet, lifestyle, stress and aging contribute to the variance in gut microbiota.^(13,15,16)^ The expansion of bacterial diversity slowly occurs in early life, as gut microbial diversity is lower in children than in adults.^(16)^ The differences in gut microbiota between the two groups were reduced in 13 weeks compared to in 8 weeks in our study. We only treated mothers; therefore, diet, housing and other environmental factors of the pups were not different between the groups. These results suggest that the differences in gut microbial composition between the two groups at 8 weeks may have been masked due to the increase of gut microbial diversity over time.

We also observed that individual differences in the gut microbiota of the mothers, especially in *Firmicutes*, were enhanced by subtherapeutic antibiotic treatment. Even at a low dose, we should consider continuous antibiotic treatments as an environmental factor and realize that they may affect host gut microbiota. Recent progress in this field showed that not only antibiotics, but also some food derivatives and food additives, affect host gut microbiota.^(17,18)^ We need to carefully study the effect of dietary components during the prenatal period on the maturation of infants’ gut microbiota and health in future life. Therefore, we should administer antibiotics thoughtfully.

In conclusion, we found that maternal subtherapeutic antibiotic exposure during the fetal period affected the gut microbiota and abdominal fat percentage of the pups without increasing body weight.

## Author Contributions

T. U., M. N., T. S., K. M. and A. T. designed research; T. U. and A. Y. conducted research and analyzed data; A. Y., T. U. and A. T. wrote paper and had responsibility for final content. All authors read and approved the final manuscript.

## Figures and Tables

**Table 1:** Oligonucleotide primer

Primer name	Sequence (5'-3')	Amplicon size (bp)	Reference
Eub338F	ACTCCTACGGGAGGCAGCAG	180	(19)
Eub518R	ATTACCGCGGCTGCTGG		
F341GC	CGCCCGCCGCGCGCGGCGGGCGGGGCGGGGGCACGGGcctacgggaggcagcag		(20)

Bact934F	GGARCATGTGGTTTAATTCGATGAT	126	(21)
Bact934GC-F	CGCCCGCCGCGCCCCGCGCCCGTCCCGCCGCCCCCGCCCGggarcatgtggtttaattcgatgat		
Bact1060R	AGCTGACGACAACCATGCAG		

Firm934F	GGAGYATGTGGTTTAATTCGAAGCA	126	(21)
Firm934GC-F	CGCCCGCCGCGCCCCGCGCCCGTCCCGCCGCCCCCGCCCGggagyatgtggtttaattcgaagca		
Firm1060R	AGCTGACGACAACCATGCAC		

Lactobacillus-F	AGCAGTAGGGAATCTTCCA	341	(22)
Lactobacillus-R	CACCGCTACACATGGAG		

Clostridia IV-F	GCACAAGCAGTGGAGT	239	(23)
Clostridia IV-R	CTTCCTCCGTTTTGTCAA		

Clostridia XIVa-F	AAATGACGGTACCTGACTAA	438–441	(24)
Clostridia XIVa-R	CTTTGAGTTTCATTCTTGCGAA		

Bacteroides-F	GAGAGGAAGGTCCCCCAC	106	(25)
Bacteroides-R	CGCTACTTGGCTGGTTCAG		
