# Supplementary material for: Effect of prenatal administration of low dose antibiotics on gut microbiota and body fat composition of newborn mice
Source: J Clin Biochem Nutr. 2017 Dec 29;62(2):155–60. doi: 10.3164/jcbn.17-53 (PMC5874232; doi:10.3164/jcbn.17-53)
Supplement: Supplemental Fig. 1 [file jcbn17-53sf01.pdf]

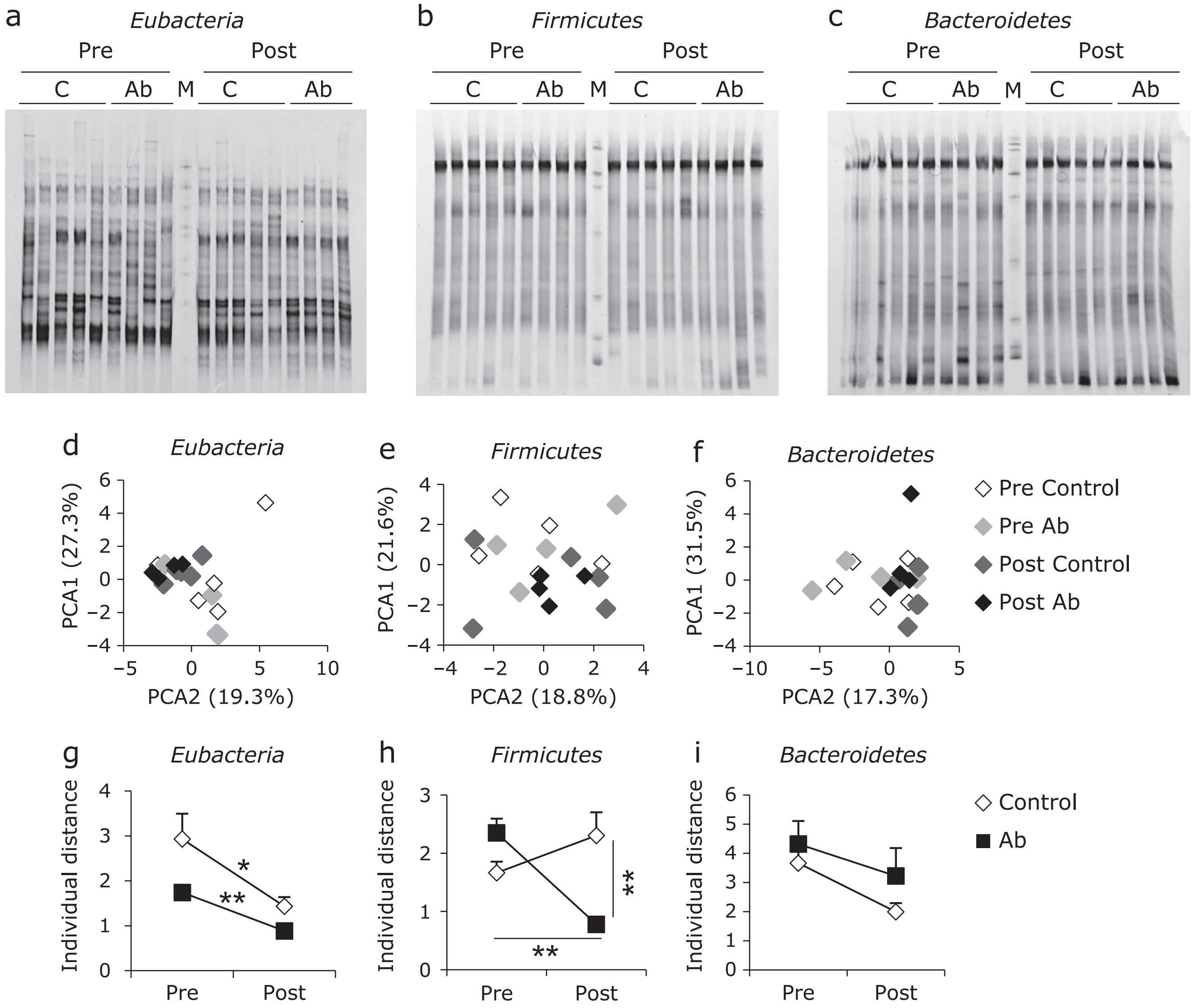

Supplemental Fig. 1. Fecal microbiota of the mother mice. (a–c) Band image of DGGE analysis of DNA from feces of mothers' total fecal bacteria (a), *Firmicutes* phylum (b) and *Bacteroidetes* phylum (c) before antibiotic administration (pre), and after antibiotic administration (post). (d–i) Two-dimensional of PCA plot of DGGE band pattern (d–f) and individual distance of PCA plot (g–i) in total fecal bacteria (d, g), *Firmicutes* phylum (e, h) and *Bacteroidetes* phylum (f, i) before antibiotic administration (pre), and after antibiotic administration (post). M, DNA marker; Control group (n = 5); Antibiotics group (Ab, n = 4). Data are shown as average and standard deviation. *p<0.05, **p<0.01.
