# Supplementary material for: Effect of prenatal administration of low dose antibiotics on gut microbiota and body fat composition of newborn mice
Source: J Clin Biochem Nutr. 2017 Dec 29;62(2):155–60. doi: 10.3164/jcbn.17-53 (PMC5874232; doi:10.3164/jcbn.17-53)
Supplement: Supplemental Fig. 2 [file jcbn17-53sf02.pdf]

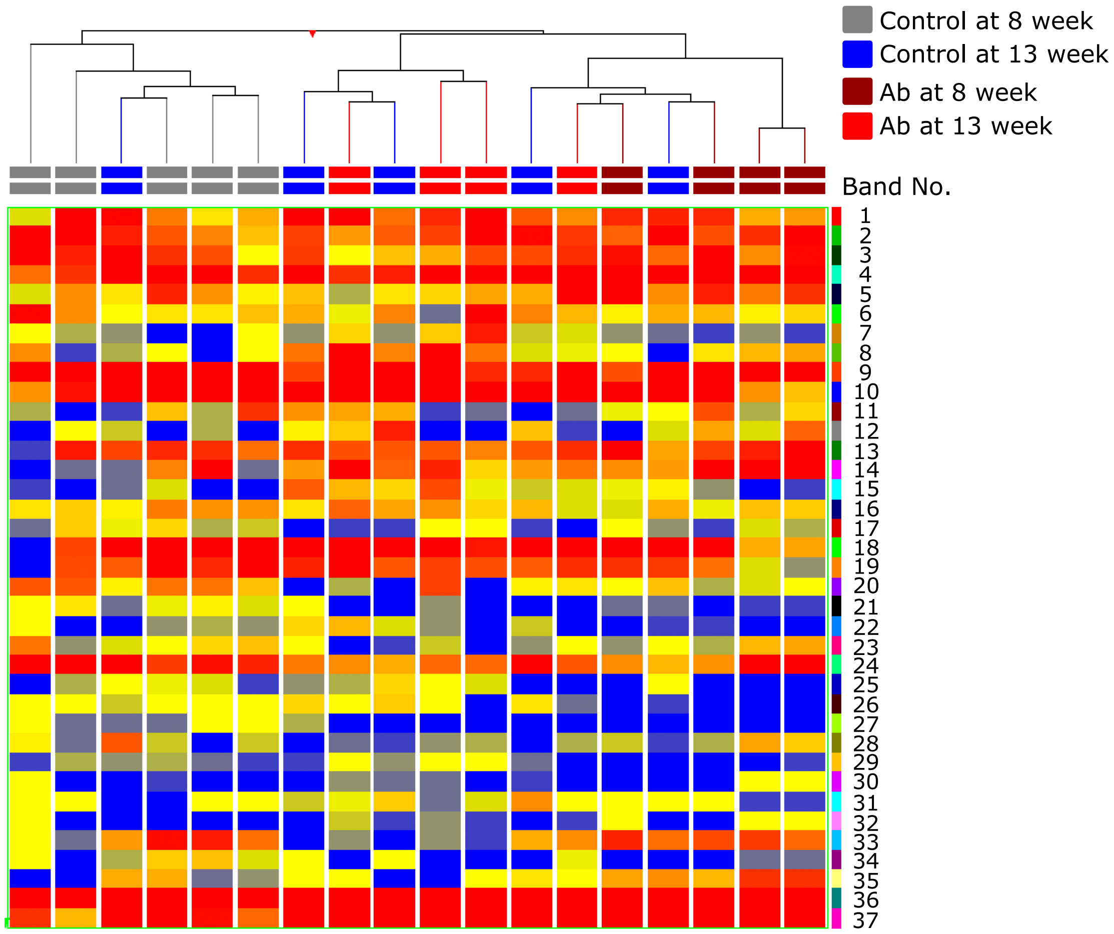

Supplemental Fig. 2. Hierarchical clustering of the gut microbiota of the pups. Hierarchical clustering of the DGGE band pattern the pups at 8 and 13 weeks. Control group (*n* = 5); Antibiotics group (Ab, *n* = 4).
