# Supplementary material for: Effect of prenatal administration of low dose antibiotics on gut microbiota and body fat composition of newborn mice
Source: J Clin Biochem Nutr. 2017 Dec 29;62(2):155–60. doi: 10.3164/jcbn.17-53 (PMC5874232; doi:10.3164/jcbn.17-53)
Supplement: Supplemental Fig. 3 [file jcbn17-53sf03.pdf]

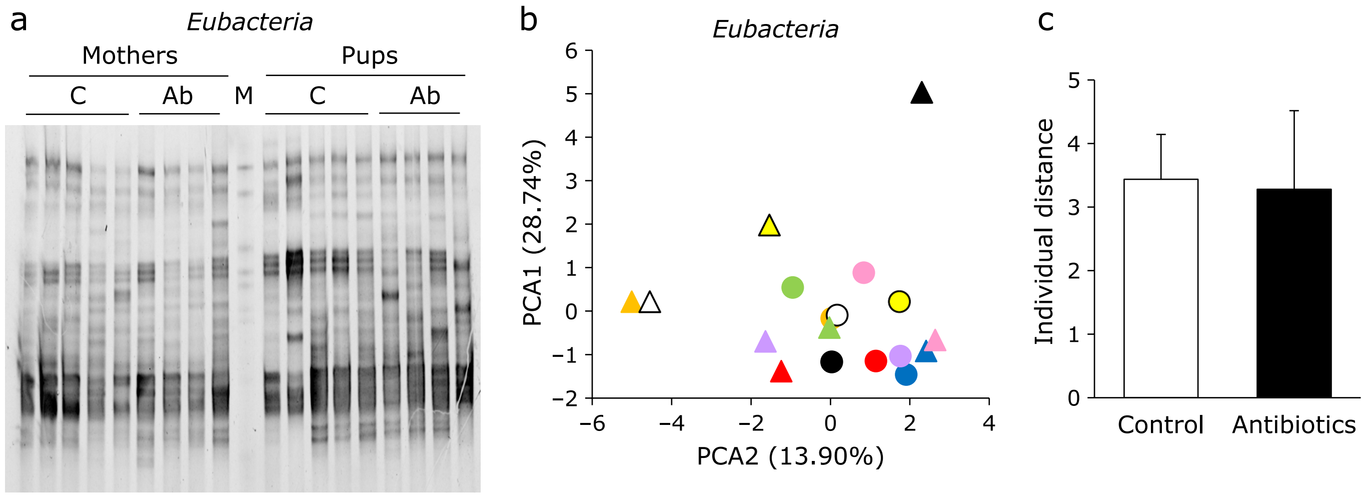

Supplemental Fig. 3. The similarity of the gut microbiota between mothers and the pups. (a) Band image of DGGE analysis of DNA from feces of total fecal bacteria of mothers and the pups (at 8 weeks). (b) Two-dimensional of PCA plot of DGGE band pattern in total bacteria (mothers: circle, pups: triangle, the same color means the pair of mother and pups). (c) Individual distance of PCA plot of the pair of mother and pups. Control group (n = 5); Antibiotics group (Ab, n = 4). M, DNA marker.
